# An in vitro evaluation of browser and grazer fermentation efficiency and microbiota using European moose spring and summer foods

**DOI:** 10.1002/ece3.3920

**Published:** 2018-03-31

**Authors:** Sophie J. Krizsan, Alejandro Mateos‐Rivera, Stefan Bertilsson, Annika Felton, Anne Anttila, Mohammad Ramin, Merko Vaga, Helena Gidlund, Pekka Huhtanen

**Affiliations:** ^1^ Department of Agricultural Research for Northern Sweden Swedish University of Agricultural Sciences Umeå Sweden; ^2^ Limnology and Science for Life Laboratory Department of Ecology and Genetics Uppsala University Uppsala Sweden; ^3^ Department of Southern Swedish Forest Research Centre Swedish University of Agricultural Sciences Alnarp Sweden; ^4^ Department of Agricultural Sciences Helsinki University Helsinki Finland; ^5^ Present address: Department of Science and Technology Høgskulen i Sogn og Fjordane Sogndal Norway

**Keywords:** bacterial community composition, browser, digestion efficiency, feed evaluation, grazer, in vitro system, methane, microbiota

## Abstract

Evolutionary morphological and physiological differences between browsers and grazers contribute to species‐specific digestion efficiency of food resources. Rumen microbial community structure of browsers is supposedly adapted to characteristic nutrient composition of the diet source. If this assumption is correct, domesticated ruminants, or grazers, are poor model animals for assessing the nutritional value of food consumed by browsing game species. In this study, typical spring and summer foods of the European moose (*Alces alces*) were combined with rumen fluid collected from both dairy cows (*Bos taurus*) and from moose, with the aim of comparing fermentation efficiency and microbial community composition. The nutritional value of the food resources was characterized by chemical analysis and advanced in vitro measurements. The study also addressed whether or not feed evaluation based on in vitro techniques with cattle rumen fluid as inoculum could be a practical alternative when evaluating the nutritional value of plants consumed by wild browsers. Our results suggest that the fermentation characteristics of moose spring and summer food are partly host‐specific and related to the contribution of the bacterial phyla *Firmicutes* and *Bacteriodetes* to the rumen microbial community. Host‐specific adaptations of the ruminal microbial community structure could be explained from the evolutionary adaptations related to feeding habitats and morphophysiological differences between browsers and grazers. However, the observed overall differences in microbial community structure could not be related to ruminal digestion parameters measured in vitro. The in vitro evaluation of digestion efficiency reveals that equal amounts of methane were produced across all feed samples regardless of whether the ruminal fluid was from moose or dairy cow. The results of this study suggested that the nutritional value of browsers' spring and summer food can be predicted using rumen fluid from domesticated grazers as inoculum in in vitro assessments of extent of digestion when excluding samples of the white water lily root, but not of fermentation characteristics as indicated by the proportions of individual fermentation fatty acids to the total of volatile fatty acids.

## 1 INTRODUCTION

Domesticated ruminants (e.g., *Bos taurus*) are adapted to utilize fibrous plant material efficiently and have a digestive system with microbial fermentation in the forestomachs characterized by selective retention of feed particles in the reticulorumen. *Bos taurus* has traditionally been considered a strict grazer, but more recent literature tends more toward considering members of the *Bovini* as more flexible, allowing for relevant proportions of browse in their diets (Clauss & Hofmann, 2014). Browsers, on the other hand, have different feeding behavior and digestive system compared with grazers, characterized by morphophysiological differences associated with the salivary glands as well as size, papillation and structure of the ruminant forestomachs (Clauss, Kaiser, & Hummel, 2008). In addition to describing feeding habitats, Hofmann (1989) pointed out the interaction between feed types and anatomical observations, and accordingly divided ruminants into three major categories: the so‐called concentrate selectors (browsers); intermediate, opportunistic, mixed feeders; and grass and roughage eaters. However, a concept of exclusiveness cannot be applied to ruminants displaying morphophysiological differences; in particular, cattle‐type digestive physiology can probably accommodate a large spectrum of diets, which would then imply a greater flexibility in feeding habitats (Clauss & Hofmann, 2014).

Clauss, Lechner‐Doll, and Streich (2003) further developed and interpreted the functional relevance of these early findings, and presented a new theory contributing to the explanation of the rumen digestive morphophysiological differences between browsers and grazers (concentrate selectors and roughage eaters, respectively, sensu Hofmann, 1989). They suggested ingesta stratification as a key factor explaining the morphophysiological differences between browsers and grazers. Ingesta stratification was assumed to promote selective retention and will hence be indicative of a more efficient utilization of fibrous plant material; the reticulorumen harboring stratified contents would be greater in size with stronger rumen pillars and with deeper reticular honeycomb cells. Further, Hofmann, Streich, Fickel, Hummel, and Clauss (2008) related the larger salivary glands of browsers to saliva protein content and viscosity, which differ from the original explanation given by Hofmann (1988) of a higher saliva production rate by browsers compared with grazers. Fermentation gases supposedly do not dissociate effectively from feed particles in a viscous medium, which results in the typical frothy appearance of reticulorumen contents of browsers (Clauss & Lechner‐Doll, 2001). This is in agreement with a generally smaller omasum, and a smaller difference between fluid and particle retention of browsers compared to grazers (Clauss et al., 2006; Dittmann et al., 2015).

Ruminants rely on symbiotic feed digestion by rumen microorganisms, which involve metabolic activities and interactions among the microbial populations that inhabit the rumen. Few studies have monitored the whole rumen microbiome of bacteria, protozoa, fungi, and archaea. Henderson et al. (2015) suggested that a core rumen bacterial microbiota occurred irrespective of host or diet, which made up two‐thirds of the community, and that diet rather than host genetics caused the main diversity changes in the other bacteria present. However, despite recent developments in sequencing technology, there is still a general paucity of data on coupled differences between grazers and browsers in terms of (1) digestion specificity and efficiency, (2) composition and structure of their rumen microbiomes, and (3) physiological characteristics of the forestomachs. In addition, to avoid confounding effects between inocula collected from either grazers or browsers in assessments of fermentation characteristics of forages and browse, it is still a norm to use domestic ruminants as a model system when assessing the value of food consumed by browsing game species (e.g., Hummel, Südekum, Streich, & Clauss, 2006). However, there is variability in the results in the literature regarding the assessment of effects of intra‐ and interspecies variability in inocula on forage and browse digestibility (e.g., Blankenship, Varner, & Lynch, 1982; Clary, Welch, & Booth, 1988; Crawford & Hankinson, 1984; Palmer, Cowann, & Ammann, 1976). In addition, the contribution to the intraspecies variability in inocula of the diet fed to the donor animals remains unclear. In this respect, the seasonal variability in the diet consumed by free‐ranging donor animals can be different from the composition of food sources to be evaluated. Further, it has been claimed impractical to make adjustment to all individual experimental dietary items in in vitro experiments suited to handle a large number of experimental treatments (Crawford & Hankinson, 1984).

The moose (*Alces alces*) is the largest species in the Cervidae family and represents a strict browser with a very low intake of monocot forage (Schwartz, 1992). During winter, the moose mainly forage on twigs of a variety of tree species (mostly pine and birch shoots). In summer, they mainly consume seedlings, leaves, forbs, and herbs (Cederlund & Nyström, 1981). It is claimed that the summer–autumn nutrition of the moose has a key role for their populations in regulating calf growth, pregnancy rates, winter survival, and body mass of the adult animals (Herfindal, Sæther, Solberg, Andersen, & Høgda, 2006). The aim of this project was to understand moose nutrition based on chemical characterization and advanced in vitro measurements with food sourced from both forest and field. The different feeds collected in spring and summer were incubated in rumen fluid of both dairy cows and moose, in order to compare the species‐characteristic fermentation efficiency in vitro and the resident rumen microbial communities. The study also addressed whether or not advanced laboratory techniques for ruminant feed evaluation based on in vitro techniques with cattle rumen fluid as inoculum are an appropriate alternative when evaluating nutritive value of browse and game field plants consumed by wild browsers.

## 2 MATERIALS AND METHODS

### 2.1 Feed samples

A set of 12 plant samples commonly consumed by the European moose were collected in Umeå (63°45′N, 20°17′E), Sweden, between 1 May and 14 August in 2012. Two samples consisted of young twigs of goat willow (*Salix caprea*) and white birch (*Betula pubescens*) representing the current season's growth of the trees. The forb fireweed (*Chamerion angustifolium*), the root of the white water lily (*Nymphaea alba*), and leaves from aspen (*Populus tremula*), white birch and rowan (*Sorbus aucuparia*) trees were collected in mid‐June and in early or mid‐August with on average 7 weeks between the sampling occasions. The samples were collected from young biotopes in clear‐cut areas, and the white water lily roots were collected in a nearby lake. Additionally, samples of red clover (*Trifolium pratense*), rape (*Brassica napus*), common vetch (*Vicia sativa*), and alsike clover (*Trifolium hybridum*) were collected from a cultivated game field in Södermanland county (58°53′N, 15°58′E) in Sweden on 21 August in 2012. After sampling, all feed samples were dried at 60°C for 48 hr. For chemical analysis and in vitro incubations, the material was ground using a stationary cutting mill equipped with a 1.0‐mm screen (Retsch SM 2000; Retsch GmbH, Haan, Germany).

### 2.2 In vitro incubations

Rumen fluid was collected from the same two fistulated nonlactating cows for all three in vitro incubations. Collection of rumen fluid from the cows was synchronized with the collection of rumen fluid from the moose. The cows were kept in a pen that housed 11 cows in total and were group‐fed to provide 5–6 kg of grass silage on a dry matter (DM) basis per animal and day. Additionally, the cows were each fed 1 kg of a commercial concentrate (Solid 220; Lantmännen Lantbruk AB, Stockholm, Sweden) on an air‐dry basis in separate concentrate feeders. The moose rumen fluid was collected from animals shot during the hunting season between 22 September and 20 October in 2012 in Vindeln (64°12′N, 19°43′E) and Robertsfors (64°12′N, 20°51′E) municipality in Västerbotten County, Sweden. Rumen fluid was collected from three different individuals and used successively in each of the three in vitro incubations. The moose shot in Vindeln municipality was an approximately 6‐month‐old female calf, and the carcass weight was 65 kg. In Robertsfors municipality, rumen fluid was collected from a 3‐year‐old bull and an 8‐year‐old cow with carcass weights of 215 and 219 kg, respectively. The rumen fluid samples from each moose were collected within 30 min after the moose was shot, immediately after the field dressing of the animals was finished. The digestive tracts from the shot moose were intact, that is, the rumen was still filled with gas, or the gut organs were not removed before our arrival in the forest. The rumen fluid from both cows and moose was filtered through two layers of cheesecloth into heated thermos flasks that were beforehand flushed with CO_2_. The feed samples were subjected to in vitro incubations where gas production was automatically recorded and corrected to normal atmospheric pressure (101.3 kPa; Cone, Van Gleder, Visscher, & Oudshoorn, 1996). Sample aliquots of 500 mg were dispensed directly into 250‐ml serum bottles (Schott, Mainz, Germany) and incubated in 60 ml of buffered rumen fluid for 96 hr. Incubations were conducted at 39°C, and the bottles were continually agitated. All samples were incubated in three consecutive runs including duplicate samples of blanks in each run.

### 2.3 In vitro sampling, analysis, and calculations

Measurement of in vitro production of CH_4_ was conducted according to Ramin and Huhtanen (2012). Gas samples from each bottle were withdrawn using a 1‐ml gas‐tight syringe (Hamilton, Bonaduz, Switzerland) after 2, 4, 8, 24, 32, 48, 72, and 94 hr of incubation. A gas volume of 22.4 L/mol, a molar mass of 16.04 g/mol, and a heat of combustion value of 55 MJ/kg were used in the calculations of CH_4_ energy losses per kg of DM.

One milliliter aliquots of rumen fluid were transferred to two replicate Eppendorf tubes after 9 and 50 hr of incubation from all bottles in every run. These samples were immediately stored at −20°C until processing for determination of bacterial community structure and volatile fatty acid (VFA) concentration. The individual and total VFA production were calculated by subtracting mean blank VFA concentration from the sample concentration. True organic matter (OM) digestibility was determined for all samples in every run from intact sample and residue composition after the 96 hr gas in vitro incubations, as described by Hetta, Cone, Gustavsson, and Martinsson (2003).

### 2.4 Chemical analysis

Residual moisture of all feed samples was determined by oven drying for 16 hr at 105°C. Ash concentration was determined by ignition of the dried sample at 500°C for 4 hr. The samples were analyzed for neutral detergent fiber (NDF) using heat stable α‐amylase and sodium sulfite by autoclaving at 105°C for 1 hr. The insoluble residue was retained by vacuum filtration in 100‐ml filter crucibles holding a porosity of 40–100 μm (Saveen & Werner AB, Limhamn, Sweden) and fitted with a glass microfiber filtering aid to trap small particles (934‐AH; Whatman Inc., Piscataway, NJ, USA). The residue was washed sequentially with hot water and acetone, and oven‐dried at 105°C for 16 hr. The NDF was expressed free of residual ash. The NDF concentration of in vitro residues was determined following the same procedure except the vacuum filtration that was replaced by centrifugation according to Udén (2006). The acid‐detergent lignin (ADL) concentration was determined by solubilization of cellulose in 12 mol/l sulfuric acid after extraction with acid detergent. The same glass microfiber filters as described above were used for the recovery of the ADL. The ADL was expressed free of residual ash. Concentrations of N were determined by Kjeldahl digestion of 1.0 g sample in 12 mol/l sulfuric acid using Foss Tecator Kjeltabs Cu (Höganäs, Sweden) in a Block Digestion 28 system (SEAL Analytical Ltd., Mequon, WI, USA) with determination of total N by continuous flow analysis using an Auto Analyzer 3 (SEAL Analytical Ltd., Mequon, WI, USA). The individual VFA concentrations were determined by high‐performance liquid chromatography (Ericson & André, 2010). The acids were separated with a packet ReproGel H column (Ammerbuch, Germany), and detected with a RI 2414 detector (Waters Assoc., USA).

### 2.5 Molecular analysis of microbial community structure

The microbial community structure was analyzed in altogether 40 samples; 32 samples corresponded to in vitro sampled rumen fluid samples pooled within feed sample and inoculum donor species; two samples corresponded to in vivo sampled rumen fluid pooled within inoculum donor species and the last six samples corresponded to the blank (rumen fluid with no sample added) in vitro flasks from all runs. Samples were processed as follows: (1) isolation of DNA; (2) PCR‐amplification of the 16S ribosomal RNA (rRNA) gene fragments performed in a two‐step procedure with the use of barcodes in the second step to enabling parallelization while minimizing PCR‐amplification bias; (3) purification and quantification of the amplified fragments; (4) finally, the samples were pooled together and sequenced using the Illumina MiSeq system (Bartram, Lynch, Stearns, Moreno‐Hagelsieb, & Neufeld, 2011).

Total DNA was isolated using 0.25 g of each sample and the Power Soil DNA isolation kit (MoBio Laboratories, Carlsbad, CA, USA) following the manufacturer's protocol. Extracted DNA was stored frozen at −20°C until further processing. Bacterial 16S rRNA gene was PCR‐amplified by adding 1 μl DNA extract to 19 μL of PCR master mix containing Phusion High‐fidelity DNA polymerase (Thermo Fisher Scientific, Waltham, MA, USA), and 1 μL of the primer‐pair 341F (5′‐CCTACGGGNGGCWGCAG‐3′)/805R (5′‐GACTACHVGGGTATCTAATCC‐3′; Herlemann et al., 2011). The 16S rRNA genes were amplified in a two‐step process as previously described (Sinclair, Osman, Bertilsson, & Eiler, 2015). For the first PCR, an initial 5‐min denaturation at 95°C was followed by 20 amplification cycles with a denaturation at 95°C for 40 s, primer annealing at 53°C for 40 s and 1‐min elongation at 72°C. Finally, a final elongation at 72°C for 7 min ended the PCR. In the second step, amplicons from the first PCR were reamplified for 10 cycles with analogous primers, except that both the forward and reverse primer featured sample‐specific 7‐bp DNA barcodes at the 5′ end. Amplicons were detected by electrophoretic separation on a 1% agarose gel followed by staining with GelRed (Biotium Inc., Fremont, CA, USA), UV‐transillumination, and image capture using a CCD camera and image analysis software (Gel‐Pro Analyzer version 3.1; Media Cybernetics, Rockville, MD, USA). Positive reactions were subsequently purified using the QIAquick PCR purification kit (Qiagen, Valencia, CA, USA) and then quantified using the Quant‐iT Picogreen assay as described by the manufacturer (Invitrogen, Carlsbad, CA, USA). Finally, samples were pooled together in equimolar amounts and sent for sequencing at the SciLifeLab SNP/SEQ facility hosted by Uppsala University, Sweden, using the MiSeq sequencing platform Illumina (Illumina, San Diego, CA, USA).

Reads were first demultiplexed based on the dual barcodes, assembled with PANDASeq, and subjected to quality control and chimera removal as previously described (Sinclair et al., 2015). Reads were then assigned into operational taxonomic units (OTUs) using Mothur (Schloss et al., 2009) according to a standard protocol (Kozich, Westcott, Baxter, Highlander, & Schloss, 2013). For this purpose, average linkage OTU clustering was applied at a 97% sequence identity cutoff, resulting in 11,569 high‐quality sequences. Detailed information about sequence analysis and annotation pipeline is given in Sinclair et al. (2015). Raw data from the 16S rRNA sequences are available in the Sequence Read Archive under the BioProject PRJNA354638.

### 2.6 Statistical analysis

The digestion characteristics derived from the in vitro system data were analyzed using the GLM procedure of SAS (SAS Inc. 2002–2003, Release 9.4; SAS Inst., Inc., Cary, NC, USA) by applying a model correcting for the effect of run, feed, species, and the interaction between feed and species. Least square means are reported, and mean separation was made by least significant difference to test differences between treatments.

The microbial composition data on the most highly resolved taxonomic level were analyzed using principal component analysis (PCA) in The Unscrambler X (Version 10.3^®^; Camo, Oslo, Norway). The presence of any systematic pattern between the samples was examined by bilinear modeling of the X matrix:

$$X=x+TP^{\prime }+E_{A},$$

where the *X* matrix is decomposed into scores of the samples in *T* and loadings for the variables in *P*ʹ, and the residuals in *E*_A_. Further, variables describing digestion characteristics were made passive, that is, they were scaled by a factor of 10^−5^ in the PCA. In this way, these variables did not influence the analysis, but could be viewed in relation to the variables describing the microbial community structure. The optimal number of principal components in the model was defined from the total residual variance (Martens & Martens, 2001). Cluster analysis was performed from the score matrix of the two‐first principal components of the PCA to provide groups of related samples. Components were joined in clusters based on Euclidean distance.

## 3 RESULTS

### 3.1 Feed sample characteristics and in vivo rumen fluid collection from moose

Chemical composition of the samples is given in Table 1. The twig samples displayed the highest concentrations in NDF and ADL, and lowest crude protein (CP) concentrations among all samples. The NDF concentration increased in botanical samples collected later in the summer, but the decrease in CP concentration between the early and late collection time was more pronounced. There was considerable variation represented in the material across feed samples; ranges in CP and NDF were from 41 to 210 g and from 169 to 595 g/kg of DM, respectively.

**Table 1:** Chemical composition of experimental samples

Feed sample^a^	No.^b^	g/kg	g/kg of DM			
		DM	OM	CP	NDF	ADL
White birch twigs	1	519	972	63	595	329
Goat willow twigs	2	486	961	74	516	221
Fireweed 1	3	172	929	77	169	36
Fireweed 2	4	271	963	95	233	61
White water lily root 1	5	87	882	86	218	58
White water lily root 2	6	106	912	41	185	51
Aspen leaves 1	7	250	943	202	252	90
Aspen leaves 2	8	401	941	120	304	101
Rowan leaves 1	9	305	935	158	206	61
Rowan leaves 2	10	394	945	66	224	79
White birch leaves 1	11	322	963	161	209	79
White birch leaves 2	12	398	958	105	317	119
Alsike clover	13	168	895	203	313	61
Red clover	14	210	907	166	365	75
Rape	15	111	869	152	354	43
Common vetch	16	256	931	210	431	90

DM, dry matter; OM, organic matter; CP, crude protein; NDF, neutral detergent fiber; ADL, acid‐detergent lignin.

a The number 1 indicates the collection time in mid‐June and number 2 the collection in early to mid‐August.

b Feed samples numbered to provide explanation to stacked columns in Figure 3.

### 3.2 In vitro fermentation and methane measurements

All measurements derived from the gas in vitro incubations are presented in Table 2. The feed × species interaction was significant (*p *<* *.01) for all traits except the TVFA and the molar proportion of butyric acid (*p *≥* *.07). Both these traits displayed significant main effects of feed and species (*p *≤* *.01). Samples incubated in moose rumen fluid generated more TVFA and a higher proportion of butyric acid than the samples incubated in cow rumen fluid (*p *<* *.01). The significant feed × species interaction indicated that the digestion efficiency of moose versus cow was substrate‐specific. Dissimilarities between ruminant species in all in vitro measured traits were mainly caused by the two samples of white water lily root. The white water lily root generated a higher gas volume, greater true OM digestibility, more CH_4_ g^−1^ of OM, and higher proportion of acetate and a smaller proportion of propionate of TVFA when incubated in moose versus cow rumen fluid (*p *<* *.01). Otherwise, acetate and propionate proportions of TVFA were generally higher and lower, respectively, when samples were incubated in rumen fluid from cow compared with rumen fluid from moose (*p *<* *.01). The results of in vitro measured true OM digestibility and end point gas volume at 96 hr were not completely consistent, except for the samples of with water lily root and birch leaves, with regard to significance of feed × species interaction. The values of true OM digestibility indicated higher ruminal digestion potential by moose than cow for the white water lily root, and the aspen and birch leaves collected in early summer (*p *≤* *.05), while the cows were more efficient in fermenting rowan leaves collected in late summer and red clover (*p *≤* *.03). In addition, the gas volume indicated higher ruminal digestion potential of birch twigs and fireweed (*p *≤* *.01) by the moose. The early collected aspen leave sample generated less CH_4_ g^−1^ of OM when incubated in moose versus cow rumen fluid (*p *=* *.02). Otherwise, the CH_4_ produced in vitro was comparable across species, except for the white water lily root that generated more than the double amount when incubated in rumen fluid from the moose compared to the cow rumen fluid (*p *<* *.01).

**Table 2:** Measurements derived from the automated gas in vitro system of all experimental feeds incubated in moose or dairy cow rumen fluid^a^

Parameter	BT	GW	FW 1	FW 2	WR 1	WR 2	AL 1	AL 2	RL 1	RL 2	BL 1	BL 2	AC	RC	R	CV	*SEM*	*p*‐Value^b^		
																		F	S	F × S
TOMD, g/kg																				
Moose	482	648	882	807	884	861	864	833	848	762	944	823	823	728	787	764	20.7	<.01	.06	<.01
Dairy cow	449	602	860	795	811	784	805	808	886	859	872	775	827	794	835	754				
CH_4_, ml/g OM																				
Moose	11.0	16.6	10.7	13.2	29.7	37.6	22.7	20.0	25.7	20.4	20.9	17.0	28.6	27.7	29.7	30.8	1.91	<.01	.28	<.01
Dairy cow	11.6	16.3	10.3	15.7	11.2	17.9	31.3	21.9	28.8	21.6	18.9	13.8	31.0	32.8	32.2	35.2				
TVFA, mmol																				
Moose	0.9	1.7	1.8	2.0	2.6	3.2	2.3	2.4	2.6	2.5	2.4	2.2	2.6	2.1	2.5	2.1	0.19	<.01	<.01	.07
Dairy cow	0.7	1.4	1.5	1.7	1.5	2.2	2.3	2.1	2.5	2.26	2.1	1.8	2.2	2.2	2.5	2.2				
AcA, mmol/mol																				
Moose	689	725	757	686	617	602	623	725	662	711	718	736	674	665	663	661	9.7	<.01	<.01	<.01
Dairy cow	717	763	798	741	566	548	725	797	735	745	760	773	730	742	732	728				
PA, mmol/mol																				
Moose	247	200	180	242	284	299	284	192	245	222	210	196	229	249	231	248	12.0	<.01	<.01	<.01
Dairy cow	199	178	147	175	355	383	180	146	155	178	190	182	184	179	177	191				
BA, mmol/mol																				
Moose	65	75	63	72	99	99	93	84	93	67	73	69	97	86	106	91	8.7	<.01	.01	.10
Dairy cow	84	59	55	84	79	69	95	57	110	77	50	46	86	79	92	82				
GV, ml/g OM																				
Moose	116	190	235	232	386	380	261	261	294	292	263	226	274	272	332	259	14.1	<.01	<.01	<.01
Dairy cow	64	151	141	178	240	280	271	231	269	266	213	192	268	260	316	269				

BT, white birch twigs; GW, goat willow; FW, fireweed; WR, white water lily root; AL, aspen leaves; RL, rowan leaves; BL, white birch leaves; AC, alsike clover; RC, red clover; R, rape; CV, common vetch; *SEM*, standard error of mean; TOMD, true OM digestibility; TVFA, total volatile fatty acids; AcA, acetic acid; PA, propionic acid; BA, butyric acid; GV, gas volume at 96 hr of incubation.

a The number 1 in feed sample name indicates the collection time in mid‐June and number 2 the collection in early to mid‐August.

b Probability of a significant effect of feed (F), species (S) and interaction between F and S (F × S).

### 3.3 Microbial community structure

**Figure 1:**
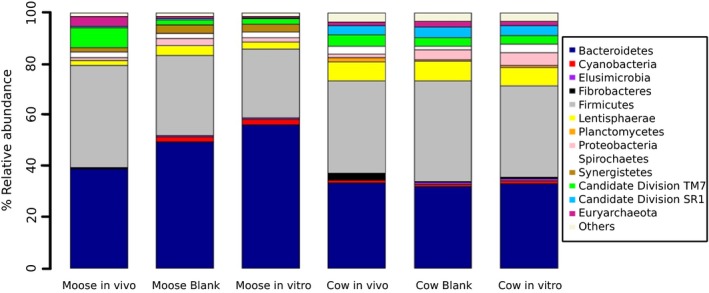
Relative abundance of bacteria and archaea at phylum level in in vivo sampled rumen fluid (*n* 3 per ungulate species), in in vitro sampled rumen fluid 9 hr after incubation start and without substrate added (blank; *n* 3) and across all in vitro sampled rumen fluid 9 hr after incubation start and with substrate added (in vitro; *n* 16) from moose and cow

The sample‐specific number of reads in the resampled data set was 11,569 sequence reads, which represent the minimum number found in any individual rumen fluid sample collected in vivo and in vitro. The majority of the 16S rRNA reads were affiliated with *Firmicutes* (40% and 36%) and *Bacteroidetes* (39% and 34%) in the in vivo sampled rumen fluid samples from moose and cow, respectively (Figure 1). For moose, the most pronounced incubation‐effect was an increase in the quantitative representation of the phylum *Bacteroidetes* when comparing in vivo to in vitro samples (increased from 39% to 50% of the total reads) mainly at the expense of *Firmicutes* (decreased from 40% to 31% of the total reads). The most pronounced change in bacterial composition for in vitro‐incubated moose rumen fluid due to the addition of feed samples was the relative abundance of *Bacteriodetes* (from 49% to 56%), and *Firmicutes* (from 31% to 27%). For cows, the bacterial community composition featured only minor differences when comparing ruminal fluid collected in vivo or observed after the in vitro incubation (Figure 1). The major change in bacterial community composition for rumen fluid from cows sampled in vitro due to the addition of feed samples was in the relative abundance of *Firmicutes* (from 39% to 36%).

**Figure 2:**
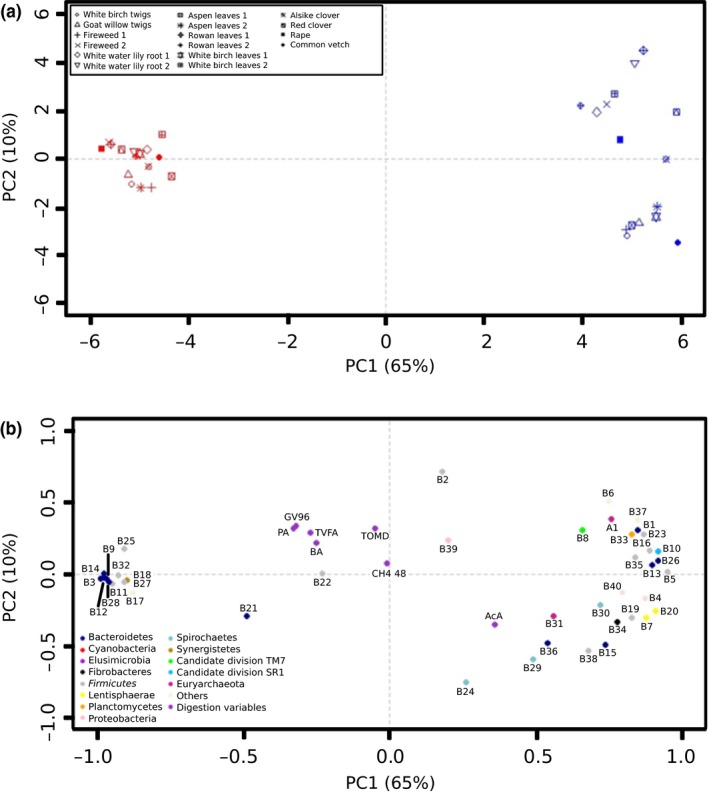
(a) Pattern of relationship between the microbial composition of rumen fluid samples (pooled between runs) from the in vitro incubations in moose (red) and cow (blue) rumen fluid in a score plot of principal component (PC)1 versus PC2. Each point represents one sample with a unique substrate added (*n* 16, see Table 1) from moose and cow. (b) Correlation loading plot of the first two PCs. Bacterial and archaeal taxa are denoted (B1, B2, ……, B40 and A1 according to Figure 3). All digestion variables in Table 2 (BA, PA, TVFA, CH_4_, TOMD) were treated as passive variables, that is, visualized and possible to interpret with the other variables, but without contributing to the explained variance by the PCs

**Figure 3:**
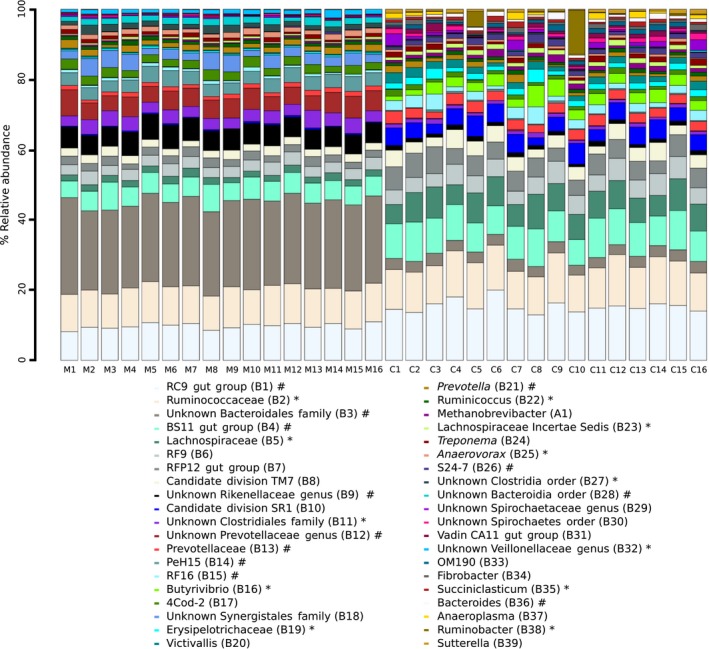
Most highly resolved taxa of the bacterial and archaeal community structure from the in vitro sampled rumen fluid from moose (M) and cow (C; samples were pooled between runs before analysis). Feed samples numbered from 1 to 16 according to Table 1. Only taxa with relative abundances >1% are given. Taxa that belong to phylum *Bacteroidetes* are labeled in the legend with # and to phylum *Firmicutes* with *

Feed samples did not group when keeping inoculum donor species as a categorical variable in the PCA (results not presented). The score plot in Figure 2a for the two‐first principal components (PC) was used to provide information about the sample distribution in the input data. There was an obvious clustering between all samples incubated in rumen fluid from cow and between all samples incubated in rumen fluid from moose (cluster distances not presented). The correlation loading plot of PC1 (abscissa) versus PC2 (ordinate) is shown in Figure 2b. To visualize all of the taxa/variables in Figure 2b, all were abbreviated B1, B2, …..B39, and A1 for most highly resolved bacterial and archaeal taxa, respectively, according to Figure 3, and in vitro digestion variables were abbreviated according to Table 2. There was only small additional variance explained from third and fourth PC (7% and 4%, respectively, to the explained variation). The loading matrix of the two‐first PC indicated highest explained variance of the moose microbial community structure by an Unknown *Bacteriodales* family (B3), an Unknown *Rikenellaceae* genus (B9), an Unknown *Prevotellaceae* genus (B12), and PeH15 (B14), and highest explained variance of the cow microbial community structure by RC9 gut group (B1), *Lachnospiraceae* (B5), RFP12 gut group (B7), Candidate division SR1 (B10), and *Butyrivibrio* (B16). Additionally, *Victavallis* (B20), *Lachnospiraceae Incertae Sedis* (B23), S24‐7 (B26), an Unknown *Clostridia* order (B27), and *Anaeroplasma* (B37) were located far on the right side of Figure 2b. The digestion variables did not contribute to any explained variance, and according to their location, they did not associate with neither the cow nor the moose microbial community structure (Figure 2b).

The microbial community structure at the most highly resolved taxonomic level of rumen fluid collected from moose and cow in vitro across all runs for all feed samples is presented in Figure 3. Generally, there were only minor differences between feed samples incubated in moose or cow rumen fluid. However, there were some differences in relative abundances of populations between the two donor species of rumen fluid. The most obvious difference was for the Unknown *Bacteriodales* family (B3) that was much more abundant across all samples incubated with ruminal fluid from moose compared to cow (22.6% vs. 2.6%). There was also a complete lack of an Unknown *Synergistal*es family (B18) and Unknown *Veillonellaceae* genus in samples incubated in cow rumen fluid, while OM190 (B33), *Succiniclasticum* (B35), Unknown *Rhodospirillales* family (B36), *Anaeroplasma* (B37), *Ruminobacter* (B38), and *Sutterella* (B39) were only present in samples incubated in cow rumen fluid.

## 4 DISCUSSION

### 4.1 Browse and game field legume samples

All plant species used in the present study have been recognized as typical components of summer diets of European browsers (Cederlund, Ljungqvist, Markgren, & Stålfelt, 1980; Holand,^1993^; Hummel et al., 2006). Aquatic plants are important diet components for the moose, but consumption is typically restricted by availability (Heptner & Nasimowitsch 1967 in Cederlund et al., 1980). Cederlund et al. (1980) concluded that trees and shrubs dominate the moose diet, comprising about half of the total diet DM between April and September. Further, most forbs occur in the diet in June and August (approximately 17%; Cederlund et al., 1980), with fireweed as a common diet species. From visual investigation of the moose rumen content from the donor animals used in the present study, it was assumed that the diet they had consumed mostly consisted of leaves from deciduous trees and shrubs.

Seasonality in the quality of the browses in the present study was reflected in the slight increase in NDF and decrease in CP concentrations in samples harvested in early to mid‐August compared to those collected in mid‐June. The twigs of birch and goat willow collected for the experiments had a diameter of up to 5 mm, which is within the range observed of moose foraging choice (Felton et al., 2016; Vivås, Sæther, & Andersen, 1991). The twig samples displayed the highest concentrations of NDF and ADL, but concentration of nonfiber carbohydrates (NFCs) would still be of the same magnitude as in the game field legumes (results not presented). Further, all game field legumes were relatively low in NDF, which suggested that the samples were regrowth material as a consequence of the late collection time and that the game had access to the fields during the summer. The browse and game field legume species used in the present study compared reasonably well in chemical composition with the species used in the study by Hummel et al. (2006).

### 4.2 Food sample fermentation and digestion efficiency

Fermentation characteristics and true digestibility measured in vitro will provide a relative ranking of the feeds, but absolute values can differ from what would have been measured in vivo due to factors like level of intake (ruminal retention of the feed), particle size, plant secondary compounds, and a lag due to adhesion of bacteria to the sample. In vitro determined true OM digestibility based on longer incubation (often more than 48 hr) in buffered rumen fluid inoculum has often been higher than in vivo determined apparent OM digestibility (e.g., Krizsan, Nyholm, Nousiainen, Südekum, & Huhtanen, 2012). This likely reflects the intrinsic and true digestibility of the feed OM in the actual species (represented by the donor animal) because there is negligible contribution of metabolic OM to undigested residues. There is a potential risk with measuring true OM digestibility in vitro between two different ruminant species with expected differences in microbial community structure and substrate‐specific rumen digestion if all NFCs have not been digested. The latter could explain the high in vitro true OM digestibility of the white water lily root in contrast to the low measured methane production when incubated in rumen fluid from dairy cows in the present study. There is also variability in the rumen fluid as inoculum within species that mostly depends on the time of collection and basal diet of the donor animal (Weiss, 1994). In the present study, the same two cows were used as donor animals throughout all in vitro runs and were kept on a similar diet the whole time. The moose inoculum, on the other hand, was different between the three runs, but could still be argued to represent a rumen fluid more adapted to summer foods than to the typical moose winter diet. Dwarf‐shrubs of blueberry and heather were likely a large component of the diets of the moose used in the present study. In the moose shot in Västmanland county in Sweden (Cederlund et al., 1980), these plant species peak as source of food in April and October (41% and 40%; in October representing equal proportion as trees and shrubs), and have their lowest occurrence in January, February, and June (between 2% and 4%). A similar pattern was found in road‐killed roe deer from southeastern Norway, used as donor animals (Holand, 1993).

In agreement with the results of Jones et al. (2001), the present study supports the use of domesticated ruminants as model animals when assessing the digestibility of food consumed by browsing game species. The significant interaction effects on the in vitro fermentation parameters in the present study rather arose due to the incubated samples of the white water lily root, which suggested an existence of a differently dietary adapted ruminal digestion in moose versus dairy cows as previously proposed by Gordon, Pérez‐Barberìa, and Cuartas (2002). When omitting the samples from the white water lily root in the statistical analysis all in vitro digestion parameters, except the molar proportions of individual fatty acids, from incubation in moose rumen fluid related well with the results when rumen fluid from the dairy cows was used as inoculum. These results were in agreement with the comparison of chamois and cattle digestion under standardized conditions of diet and passage rate made by Dalmau, Ferret, Manteca, and Calsamiglia (2006). Further, the fermentability was numerically higher in rumen fluid from the moose compared with the cow for all samples, except when incubated with rowan leaves and the game field legumes. This may suggest lower abundance of secondary compounds in leaves from rowan or a different composition of such metabolites compared with the other browse as reported by Makkar, Blümmel, and Becker (1995). Our results further suggested that concentrations of secondary compounds in browse leaves could have had a smaller effect on the degradation when samples were collected later rather than earlier in the season. This is in agreement with the observations by Singh, Sahoo, Sharma, and Bhat (2005) that fiber composition will be of greater significance to the ruminal digestion than plant secondary compounds. Additionally, the represented game field legumes in the present study were judged to be a valid food source for moose since fermentation compared well in absolute values to browse, especially regarding the indication of seasonality in feed quality represented in this material. Higher asymptotic gas volumes of grass silage samples have correlated well with a higher feed intake by growing cattle (Krizsan, Nyholm, et al., 2012), and in vitro digestibility of OM has been positively related to forage quality and intake by domestic ruminants (Krizsan, Hetta, Randby, & Huhtanen, 2012).

A fundamental principle in nutritional ecology of herbivores is that diet choices are related to digestion efficiency and that food habits reflect morphophysiological adaptations to assimilate nutrients by the animal. Generally, it is assumed that rumen fill capacity and intake are intimately coupled. However, Holand (1994) pointed out the theory of phenotypic plasticity of browsers, which involves regulation of ruminal retention of digesta, depending on feed quality and quantity. The ad libitum intake of concentrate by roe deer was higher in summer than in winter, and resulted in a significantly accelerated rate of passage rather than an increase in gut fill. This change in rate of passage did not affect total tract apparent digestibility. These results confirmed that roe deer behaved as small‐bodied browsers adapted to high intake, rapid turnover, and rapid digestion, when high‐quality feed sources were available. Further, the ad libitum intake and the rumen fill were seasonally stable for roe deer fed blueberry shrubs. Comparing roe deer fed blueberry shrubs ad libitum versus restrictively resulted in faster propulsion of the digesta through the system, keeping the fill rather constant. Further, Holand (1994) suggested that food availability rather than the rumen capacity limit the voluntary intake by browsers during the winter season. The rate of passage of digesta is then downregulated to keep a viable and stable rumen environment, and to minimize energy deficiency. The nutritional strategy of browsers thereby includes the uncoupling of rumen fill from intake, but the mechanism remains unknown. Using available, comparable data on fluid and particle retention in ruminants, Dittmann et al. (2015) demonstrated that results indicate a comparatively longer particle, but a shorter fluid retention in the forestomach of cattle as compared with either giraffe, okapi, or moose. In other words, grazers and intermediate feeders retain particles longer in their reticulorumen per unit fluid retention time than browsers, and that cattle are exceptional in this respect with very long particle retention times per unit fluid retention. Based on these results, but in contrast to Hummel et al. (2015) and Dittmann et al. (2015), we speculate that less feed energy is used for microbial maintenance in the moose compared with the dairy cow, but that they still need to selectively forage on browse high in protein during summer to balance the high availability of rapidly digestible carbohydrates.

Considering the high‐feed quality of most of the summer browse the shift in the proportions of acetate and propionate between incubation of samples in rumen fluid from the two different ruminant species in the present study was not unexpected. A shift in ruminal fermentation pattern with decreased acetate to propionate ratio is consistent with what would be expected from increased carbohydrate fermentation. Dalmau et al. (2006) observed no difference in the total extent of digestion between chamois and cattle in vitro, but differences in fermentation pattern represented by the individual VFAs. They suggested that the microbial populations that inhabit the rumen of these animals have the same capacity to digest, but with different fermentation profiles and different microbial protein synthesis. The fireweed and the white water lily root were among all samples in the present study highest in concentration of NFCs (results not presented). However, the plants induced completely different patterns of fermentation in the in vitro incubations compared to the species characteristics otherwise found for rumen fluid from moose versus cow. The major carbohydrates in the white water lily root are likely different from that in the fireweed causing the observed differences in fermentation pattern between the two samples. Van Soest (1994) pointed out that the Arctic ruminant group (e.g., reindeer and moose) must shift their feeding from browse in summer to lichens or wood in winter. In line with the moose feeding on water lily root, lichens play a specific role in the nutrition of reindeers. However, due to the required adaptation of rumen microbes in Arctic ruminants to digest lichenin (a type of beta glucan) or wood, Van Soest (1994) suggested they should not be classified as pure browsers.

The in vitro method used in the current study has previously been used for determination of methane from various feed samples. Browse high in NFCs is traditionally regarded to lower methane production by stimulating propionate production relative to the other fermentation acids or by antimicrobial effects of plant secondary compounds in browse. White and Lawler (2002) did not observe increased production of CH_4_ in muskoxen fed an increasing proportion of leafy browse in the diets. They pointed out a different response from diets containing woody browse and explained lack of positive relationship between amount of leafy browse and CH_4_ production by a generally higher production of CH_4_ from the leafy versus woody browse. This is in agreement with the results obtained in the current study. Further, a noteworthy high amount of CH_4_ was produced when the white water lily root was incubated in moose rumen fluid, again emphasizing the deviating properties of this feed compared to the others in the present study. The global estimation of CH_4_ production from wild animals such as the moose is difficult due to the lack of sufficient data on animal population, intake, and food digestion. The proportion of CH_4_ to the gross energy intake is typically between 6% and 7% in dairy cows, but it can vary from 2% to 12% depending mainly on the type of diet and physiological stage of the animal (Johnson & Johnson, 1995). On an average level of all feeds used in the current study, the proportion of CH_4_ to the gross energy intake was estimated to be 5.0% and 4.8% for moose and cow, respectively. Yan, Agnew, Gordon, and Porter (2000) reported a value of 6.1% CH_4_ as a proportion of gross energy for dairy cows at the production level. The lower levels found in the present study could be related to the uncommon feeds used in the current study with those typical diets fed to dairy cows (e.g., grass silage). The very low CH_4_ values produced for some feeds used in the current study (e.g., fireweed) could also indicate a potential inhibitory effect on CH_4_ production, especially when comparing the values of CH_4_ produced to VFA production.

### 4.3 Microbial community structure

The recent development of high‐throughput sequencing techniques, such as Illumina, has increased our understanding of microbial community composition and enabled in‐depth analyses of microbiomes in a wide range of environments. The ruminant gut is no exception (McCann, Wickersham, & Loor, 2014). Previous studies about the gut microflora in domesticated ruminants have focused on finding suitable CH_4_ mitigating strategies and estimating the efficiency of feed utilization by targeting archaeal members as those actually mediating methanogenesis. However, about 95% of the bovine rumen microbial community consists of bacteria that play key roles in preprocessing OM for methanogen use (Brulc et al., 2009). These bacterial communities are likely to change with the feed, and our study contributes to increase our knowledge about the ruminant microbiome by comparing bacterial composition in the rumen from one browser and one domestic species (grazer‐mixed feeder).

Henderson et al. (2015) studied the rumen microbial community in 32 different species of ruminants from 35 countries and concluded that the bacterial community contributed to the main observed differences among the species. In addition, they defined the common core ruminant microbiome, with members from *Bacteroidetes* and *Firmicutes* comprising around 70% of the sequences with *Prevotella*,* Butyrivibrio*, and *Ruminococcus* being the most abundant at genus taxonomic level. That is in agreement with earlier studies also reporting on *Bacteroidetes* and *Firmicutes*, being the most abundant phyla in rumen samples (Jami & Mizrahi, 2012; Mandal, Saha, & Das, 2015).

In our results, microbial communities exhibited contrasting patterns depending on the host species, but were highly similar among individuals from the same host species. Change in relative abundances among hosts has been observed in previous studies (Jami & Mizrahi, 2012) and can be mainly explained by different dietary adaptations in the regulation of the microbial communities present in the rumen (Henderson et al., 2015). On the other hand, the small differences observed in microbial community composition of the rumen between individuals within the same host species can be explained by a combination of both genetic and environmental factors. Most likely, part of the microbial community is hereditary as closely related individuals are known to host fecal communities that are more similar compared to more distantly related hosts (Reyes et al., 2010). In addition, it is known that environmental factors such as age, diet, rumen temperature, rumen pH, location among others can also influence the rumen microbial composition (Ishaq et al. 2015; Mandal et al., 2015). Earlier work has shown high similarity of cow gut microbiota at different times and locations (Li, Penner, Hernandez‐Sanabria, Oba, & Guan, 2009). However, Henderson et al. (2015) observed that changes in microbial communities seemed to depend on diet, as animals with forage‐dominated diets were more similar to each other, with a higher abundance of members from family *Ruminococcaceae*, while animal with concentrate dominated diets featured higher abundance of members from genus *Prevotella*, and were thus distinct from the foraging animals. Therefore, it seems that diet and host are the most significant factors determining microbial community composition.

The link between bacterial components of the rumen microbiome and its role in the host animal is difficult to uncover as the functionality of many bacterial groups is still not well understood (Henderson et al., 2015). In our results, *Bacteroidetes* and *Firmicute*s were well represented in samples from moose and cow rumen fluid at the most highly resolved data. Within *Bacteroidetes*, some of its members are known to hydrolyze polysaccharides present in cells walls, thus assisting the host with the degradation and fermentation of the OM (Liu, Zhang, Zhang, Zhu, & Mao, 2016). The two most abundant *Bacteroidetes* families were as follows: (1) *Ruminococcaceae* with members known to perform polysaccharide and fiber degradation for downstream nutritional needs, and (2) *Prevotellaceae*, with *Prevotella* as the most abundant genus of this family, known to use proteins and carbohydrates provided in the diet. Those two families have been also found to be highly abundant in previous studies (Henderson et al., 2015; Jami, Israel, Kotser, & Mizrahi, 2013; Liu et al., 2016). Within *Firmicutes*, family *Lachinospiraceae* was the most abundant, especially in cow rumen samples, with *Butyrivibrio* being the most representative genus indicating an increase in butyrate availability in the rumen of cows compared to the moose. In addition, other genera identified in our samples, such as *Treponema,* are known to play a role in cellulose digestion (Liu et al., 2016). *Succiniclasticum* was only found in cow rumen samples and are believed to be involved in fiber degradation and propionate formation (Henderson et al., 2015; Liu et al., 2016).

The abundance of some bacterial taxa was also associated with host lineage as unclassified *Veillonellaceae* were more abundant in cervids and caprids than bovines, while *Fibrobacter* were more abundant in bovines and may play an essential role in the degradation of plant fiber in cattle (Henderson et al., 2015). In agreement with this, we found an Unknown *Veillonellaceae* genus only in rumen fluid of moose, and *Fibrobacter* were much more abundant in the samples of rumen fluid from cows. Although modern sequencing technology generates a large amount of information about the complex microbiota inhabiting natural systems such as the rumen, more detailed studies targeting specific groups are needed to increase our understanding about the specific functional role of microbial community members in the rumen.

## 5 CONCLUSIONS

The results of the present study suggest that the ruminal fermentation in vitro of the main spring and summer food for moose cannot distinguish between whether the ruminant species used as inocula door animal is a browser or grazer within the limitations of one species of each as representatives. The interactions between feed and ruminant species clearly indicate that rumen fluid from dairy cows should not be used to rank food resources with regard to establishing the nutritional value of browse. Our results suggest that the species specificity of moose and dairy cow micro flora was associated with marked differences in ruminal microbial community structure of the bacterial phyla *Firmicutes* and *Bacteriodetes*. Host animal‐specific ruminal microbial community structure is in agreement with the concept of evolutionary adaptations related to feeding habitats, morphophysiological differences, and ruminal retention of digesta (i.e., physiological features of energy‐harvesting abilities) between browsers and grazers. However, the observed differences in microbial community structure could not be related to ruminal digestion parameters measured in vitro. There was a shift in ratios of VFAs in vitro depending on donor species inoculum that also could be related to the substrate, that is, the chemical composition of the browse. A larger population of game field plants and plants collected at different time points throughout the whole season needs to be evaluated to be able to more robustly compare the game field plant nutritive value to the summer food preference by the moose. However, the represented game field legumes in the present study can be regarded a valid food offer to the moose since chemical composition and fermentation parameters compared well in absolute values to browse, especially regarding the indication of seasonality in feed quality represented in this material.

## CONFLICT OF INTEREST

None declared.

## AUTHORS' CONTRIBUTIONS

SJK, PH, AMF, and SB conceived the ideas and designed methodology. SJK, AMF, SB, AMR, AA, MV, MR, and HG collected the data. SJK and PH analyzed the data. SJK led the writing of the manuscript. All authors contributed critically to the drafts and gave final approval for publication.

## DATA ACCESSIBILITY

All relevant data of the present study are in Dryad. The raw reads are available in the Sequence Read Archive hosted by NCBI.
